# Impedance-guided Radiofrequency Ablation: Using Impedance to Improve Ablation Outcomes

**DOI:** 10.19102/icrm.2017.081003

**Published:** 2017-10-15

**Authors:** Jason S. Chinitz, Gregory F. Michaud, Kent Stephenson

**Affiliations:** ^1^Department of Cardiology, Hofstra Northwell School of Medicine, Northwell Health, Southside Hospital, Bay Shore, NY, USA; ^2^Arrhythmia Section, Vanderbilt Heart and Vascular Center, Vanderbilt University Medical School, Nashville, TN

**Keywords:** Atrial fibrillation, catheter ablation, impedance, impedance decrease, lesion assessment

## Abstract

Despite the achievement of acute conduction block during catheter ablation, the recovery of conduction at previously ablated sites remains a primary factor implicated in arrhythmia recurrence after initial ablation. Real-time markers of adequate ablation lesion creation are needed to ensure durable ablation success. However, the assessment of acute lesion formation is challenging, and requires interpretation of surrogate markers of lesion creation that are frequently unreliable. Careful monitoring of impedance changes during radiofrequency catheter ablation has emerged as a highly specific marker of local tissue destruction. Ablation strategies guided by close impedance monitoring during ablation applications have been demonstrated to achieve high levels of success for ablation of atrial fibrillation. Impedance decrease during ablation may therefore be used as an additional endpoint beyond acute conduction block, in order to improve the durability of ablation lesions. In this manuscript, available methods of real-time lesion assessment are reviewed, and the rationale and technique for impedance-guided ablation are described.

## Introduction

The technical goal during catheter ablation targeting an arrhythmogenic substrate is the creation of durable transmural tissue destruction at precise myocardial locations. In order to achieve effective ablation delivery, adequate tissue contact, catheter stability, and good lesion time and power are required. Advancements in catheter technology now allow users to achieve substantially greater control and awareness of catheter–tissue contact.^1^ However, despite the greater control of these factors involved in energy delivery, determining whether adequate tissue injury has occurred during ablation is still imprecise, given the inability to directly assess lesion formation during routine ablation procedures.

Even when acute ablation success is achieved, arrhythmia recurrence is frequently a result of recovery of conduction in incompletely damaged myocardial tissue. The recovery of conduction following initially successful ablation may be the result of the creation of incomplete and therefore reversible myocardial injury or edema formation in regions adjacent to ablation lesions. Both situations may mask the need for further ablation to achieve permanent electrical inactivity, since the stunned tissue may not be distinguishable from tissue that will form a permanent scar.

Real-time markers of adequate ablation lesion creation are clearly needed to guide effective ablation application. However, despite our improving ability to titrate energy input, the assessment of ablation effects is challenging, and requires the interpretation of surrogate markers of lesion creation that are frequently unreliable. Careful monitoring of impedance changes during radiofrequency (RF) catheter ablation has emerged as a highly accurate marker of local tissue destruction. Impedance decrease occurring as a result of catheter ablation has been correlated with lesion formation, and may be the most reliable acute marker of tissue injury at a given target site. Ablation strategies based on ensuring impedance fall during ablation applications have been demonstrated to achieve high levels of success for the ablation of atrial fibrillation (AF),^2^ and sites of recovered conduction have been closely correlated with inadequate impedance decreases observed during initial ablation.^3^ In this manuscript, the available methods of real-time lesion assessment are reviewed, and the rationale and technique for impedance-guided ablation are described.

## RF lesion assessment

RF energy produces controlled lesions at the catheter tip using resistive heating, with convective heat conduction traveling to deeper tissue, resulting in coagulation necrosis and local lesion formation. Many factors impact the size and depth of ablation-induced tissue destruction. Some of these factors are controllable, such as power delivery, duration of energy application, degree of contact force, use of irrigation, catheter orientation, and the electrode radius of the ablation catheter used; however, other factors that are uncontrollable, including tissue characteristics such as wall thickness, the presence of trabeculations or fibrosis, and/or thoracic or cardiac motion that affects catheter stability, also play important roles in effective lesion creation. Accordingly, power input and other ablation parameters needed to achieve transmural lesions are variable, and markers of true lesion formation are needed to guide ablation delivery.

The direct measurement of tissue temperature is commonly performed, and in some circumstances may be used as a surrogate of lesion formation. To achieve irreversible tissue injury, a tissue temperature of 50°C to 58°C is required, whereas ablated tissue of temperatures of less than 50°C often results in tissue stunning and reversible tissue injury. During ablation with non-irrigated catheters, catheter tip temperature can be directly measured, and the maximum temperature reached within the tissue correlates linearly with lesion volume;^4^ however, the temperature at the tissue interface is much higher than the measured electrode temperature. When the temperature at the catheter–tissue interface exceeds 80°C, which often happens when higher power is used, there is a propensity for coagulum formation on the catheter, which results in a rise in local impedance, limiting RF current delivery and lesion formation. As a result, saline irrigation is now routinely incorporated into ablation catheters to maintain a low electrode–tissue interface temperature during RF application at high power, allowing for more energy application without coagulum formation. However, active irrigation renders the temperature measured at the catheter tip to be completely non-reflective of deeper tissue temperatures.^5^

**Figure 1:**
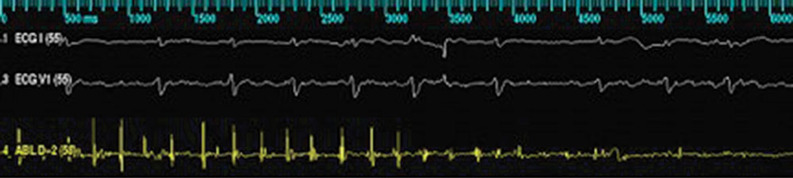
Diminution in EGM amplitude during ablation. A bipolar EGM measured from the ablation catheter tip during ablation is shown in yellow, and surface electrocardiogram is shown in white. Note the change in EGM amplitude during ablation.

Another marker of tissue injury commonly used to guide ablation delivery is electrogram (EGM) amplitude diminution. A decrease of bipolar EGM amplitude of 80% at the site of ablation, compared with pre-ablation amplitude, has been associated with the creation of a transmural lesion in animal models **(Figure 1)**.^6^ Similarly, the creation of a completely positive unipolar signal has also been associated with transmural lesion formation, and achievement of this endpoint has been associated with improved outcomes after AF ablation.^7^ However, the assessment of real-time EGM diminution is difficult to quantify in real time, and can also be misleading, as changes in catheter position and catheter–tissue contact can alter EGM morphology and amplitude. Indeed EGM diminution can reflect either actual tissue destruction or, simply, the absence of adequate contact between the ablation catheter and the tissue. EGM diminution is therefore a non-specific marker and is frequently unreliable during physiologic conditions.

The assessment of pace excitability is an additional method useful for assessing adequate lesion creation. Following or during ablation, pacing can be performed over the region of ablation to ensure a lack of pace capture, thereby inferring local tissue destruction. This method may be useful in the identification of incompletely damaged tissue along the lines of ablation, and may guide the decision regarding the need for further ablation.^8,9^ An AF ablation strategy confirming non-excitability along the line of ablation has been shown to improve AF ablation outcomes and reduce the risk of AF recurrence beyond electrical pulmonary vein isolation (PVI) alone.^10^ However, the assessment of pace excitability cannot reliably distinguish tissue that has been permanently scarred from stunned tissue that will eventually recover conduction capabilities. Furthermore, as pacing cannot be easily performed during ablation with certain ablation technologies, this technique may require an additional step following the completion of ablation that may prolong the procedure time and complexity. Better real-time and specific markers of durable tissue destruction are needed to improve ablation success.

### Impedance change with ablation

Impedance decrease during RF ablation is a specific marker of local tissue heating, and therefore can be used as a real-time indicator of lesion creation. Local impedance changes occur during RF ablation as the tissue temperature rises, resulting in increased ion mobility within the tissue being heated, and therefore a decrease in impedance to current flow.^11^ If the myocardium interface is not adequately heated—regardless of whether such is a result of poor tissue contact, lack of catheter stability, or inadequate power delivery—the characteristic decreases in impedance measurements for a given catheter location do not occur. Lesion diameter and depth have been shown to correlate well with impedance decrease, and a more direct correlation to lesion depth has been shown with impedance decrease than with temperature achieved with both irrigated and non-irrigated ablation.^12,13^ Prior clinical and experimental studies have found an impedance decrease from baselines of at least 5 Ω to 10 Ω to be best associated with effective tissue destruction, regardless of the baseline tissue characteristics.^12,14,15^ Impedance monitoring can also guide the duration of lesion application, as lesion expansion may continue for 10 s following reaching the plateau of the impedance curve.^16^ Additionally, impedance monitoring has an additional important role for monitoring safety of RF energy application, as steam pops are rarely seen when the impedance decrease is less than 15 ohms (Ω).^13,17^

Clinical data have supported the utility of performing PVI guided by close impedance monitoring with a standard RF ablation catheter (non-contact force-sensing). By ensuring an impedance decrease of at least 5 Ω in the first 10 s of ablation, 84% of patients with paroxysmal AF remained free of demonstrable recurrent atrial arrhythmias after 431 ± 87 days.^2^ Furthermore, in a retrospective analysis of repeat ablations for recurrent atrial arrhythmias among patients who underwent point-by-point circumferential pulmonary vein (PV) antral ablation, 89% of recovered PV conduction occurred in specific regions in which the impedance decrease was < 10 Ω during initial ablation.^3^ In addition, regions with adjacent ablation resulting in impedance decrease < 10 Ω were associated with a higher rate of conduction recovery (37% versus 1.5%; p < 0.001). In contrast, small visual gaps between lesion markers had no correlation with PV conduction recovery in this study, assuming the surrounding ablation applications resulted in > 10 Ω impedance decrease. Though not all sites with lesser impedance decreases correlated with PV conduction recovery, conduction recovery was rare in the presence of an impedance decrease of > 10 Ω, and occurred in only two patients in whom reconnection was found in the interatrial septum along the anterior aspect of the right PVs, where the thickness of atrial myocardium can be substantial. These findings indicate that an impedance decrease ≥ 10 Ω during RF delivery may represent a specific marker of durable lesion formation in thin atrial tissue, allowing an operator to alter modifiable factors such as contact force, catheter orientation, or power, in order to achieve this endpoint.

## Role of contact force

The routine use of contact force-sensing catheters now allows for the optimization of the catheter–tissue interface, and has improved both clinical outcomes and procedure safety.^1^ Lesion size correlates with contact force and contact time, and validation in animal models has shown a linear correlation between adequate contact and lesion transmurality, despite constant power and identical peak contact forces.^18^ As contact force is an important factor in lesion creation, increased catheter–tissue contact has now been associated with a larger impedance decrease during RF ablation,^12,17,19^ and in the absence of sufficient catheter–tissue contact, an impedance decrease of 10 Ω cannot be achieved.^12^

However, contact force is only one of several factors associated with lesion creation (ablation power, duration, surface area, catheter stability and tip-to-tissue contact, and local tissue resistance being other examples) and, when measured in isolation, does not indicate effective tissue destruction. Furthermore, the measurement of contact alone may actually be misleading when recorded contact force is measured from the catheter shaft rather than from the tip, or in cases in which poor stability results in variable contact during ablation. Accordingly, one study found a significant difference in impedance decrease, but not contact force or force–time integral, between adequate and inadequate ablations.^20^ Since contact force is modifiable prior to ablation delivery using available force-sensing catheters, applied contact may be considered to be an important predictor of lesion creation, though it is not a marker of tissue heating or an assurance of adequate lesion formation. In contrast, a resulting impedance decrease of > 10 Ω indicates that such heating has occurred, and that a durable lesion is likely.^3^

## Performing impedance-guided ablation

**Figure 2:**
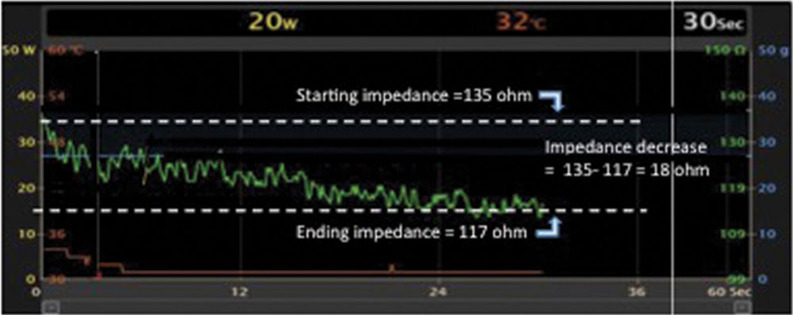
Graphical representation of impedance decrease during ablation, with impedance measurement (green) presented on the Y-axis and time presented on the X-axis. The Y-axis is set up with a range of 50 Ω (100 Ω to 150 Ω in this example) centered around the starting impedance value. Dashed white lines represent starting and ending impedance values, and the decrease in impedance during ablation (starting impedance minus the ending impedance) is calculated.

An accurate interpretation of impedance changes during ablation requires the designation of a point-by-point ablation application strategy, along with the making of efforts to avoid “dragging” lesions at consecutive sites without interruptions of ablation. The described meticulous impedance monitoring requires the creation of a dedicated graph that displays real-time impedance on the mapping system, with impedance on the Y-axis and time on the X-axis. In order to accurately evaluate even small impedance changes with ablation, we generally change the scale on this graph to a range of 50 Ω, centered around the starting impedance **(Figure 2)**. While the CARTO3 system (Biosense Webster, Diamond Bar, CA, USA) had previously been the primary mapping system that permitted such manipulation of the impedance display, other available mapping systems are now adding this capability. The delta impedance is then calculated as the starting impedance—the lowest impedance measured during RF ablation in a single catheter location.

**Figure 3:**
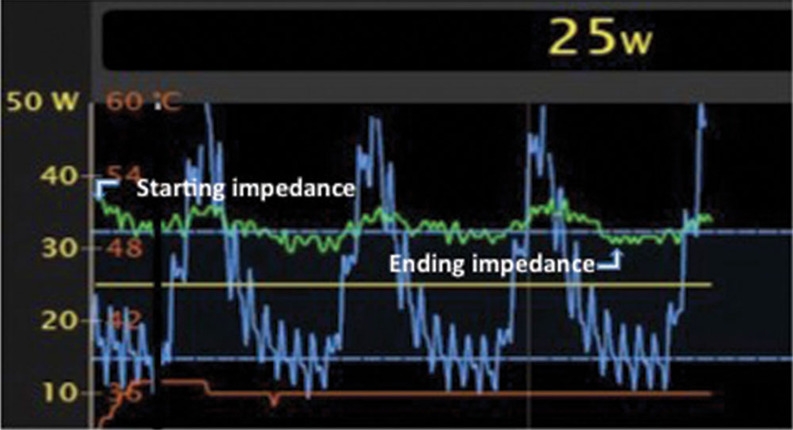
Graphical representation of both impedance and force variation during ablation, with impedance measurement (green) and contact force (blue) presented on the Y-axis and time presented on the X-axis. Note the cyclical changes in both force and impedance occurring with the change in the respiratory cycle. The starting and ending impedance measurements are taken during the same phase of respiration (expiration in this case).

**Figure 4:**
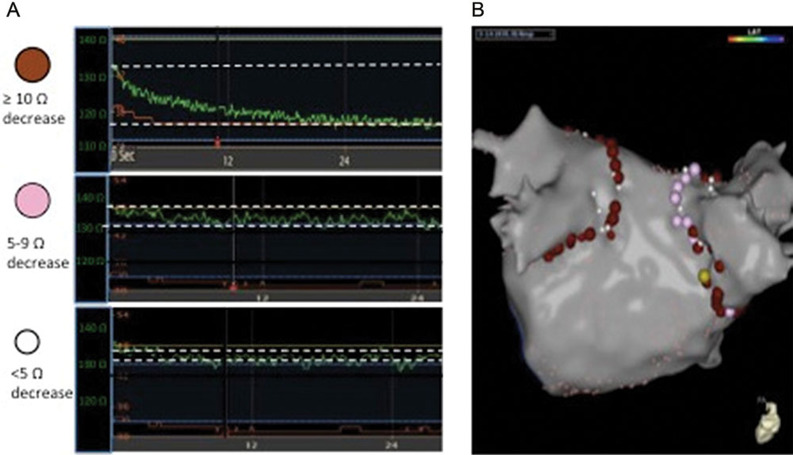
**A:** Ablation markers applied on the electroanatomic map are color-coded indicating the magnitude of impedance decrease with ablation. The red markers indicate ≥ 10 Ω impedance decrease, the pink markers indicate a 5 Ω to 9 Ω decrease, and the white markers indicate a < 5 Ω decrease. In the presented graphs, the impedance during ablation is shown in green, with an impedance scale (green text) presented on the Y-axis and time presented on the X-axis. Dashed white lines represent starting and ending impedance values. **B:** An electroanatomic map with color-coded ablation markers denoting various impedance decreases as were described in **A**.

Fluctuations in impedance relating to respiratory motion are accounted for by using impedance values measured in the same phase of the respiratory cycle at the start and end of ablation application **(Figure 3)**. Ablation lesion markers can then be color-coded based on the impedance decrease achieved during ablation, in order to document achieved impedance changes and direct additional ablation efforts as necessary. In our electrophysiology laboratory, a red ablation marker represents a ≥ 10 Ω impedance decrease in ≤ 30 s of ablation, while a pink lesion marker represents an impedance decrease of 5 Ω to 9 Ω and a white marker represents an impedance decrease of < 5 Ω **(Figure 4)**. Efforts can then be made to terminate low-impedance lesions (< 10 Ω) and reorient the catheter to achieve better tissue contact.

### Limitations of impedance-guided ablation

The accurate interpretation of impedance changes with ablation requires a point-by-point ablation strategy, as catheter movement during ablation will alter local impedance measurements. Respiratory variation, which is typically present during ablation in the absence of forced apnea or jet ventilation, can also alter impedance measurements during ablation, and thus should be assessed and accounted for prior to deciding that adequate impedance decrease has occurred. Therefore, mapping systems that do not allow the operator to alter the impedance scale typically preclude an accurate interpretation of impedance trends.

While an impedance fall of at least 10 Ω is a specific marker of tissue heating, the sensitivity of this marker may be limited, as ablation applications resulting in lesser impedance decreases may still result in transmural lesion creation. In thin areas of atrial tissue, catheter tip irrigation may directly impact the impedance decrease with ablation by sparing the endocardial surface of direct heat delivery, thereby making the measured impedance change less reflective of lesion progression at deeper tissue levels. Though impedance measurements at the catheter tip may be influenced by several factors beyond local tissue characteristics, the decrease in impedance during RF ablation is almost entirely due to variations in local tissue impedance, and thus the delta impedance remains a useful measure independent of most interpatient variables.

## Conclusions

Despite the achievement of acute arrhythmia suppression or conduction block during RF ablation, the recovery of conduction at previously ablated sites remains a primary factor implicated in arrhythmia recurrence after initial ablation. Real-time markers of adequate lesion creation with ablation are needed in order to guide ablation delivery and reduce arrhythmia recurrence after ablation. An impedance decrease of ≥ 10 Ω indicates local tissue heating has occurred, perhaps mostly at the catheter–tissue interface, and is a specific marker for durable ablation success, at least in thin atrial tissue. Impedance changes can therefore guide adjustment of ablation input parameters (including power, duration, and tissue contact) until an adequate impedance decrease is achieved.

Impedance decrease over 10 Ω may be used as an additional endpoint beyond acute conduction block, in order to improve the durability of ablation lesions, and prevent recurrence.
